# Neuroborreliosis with severe mental confusion and thickening of cranial nerves

**DOI:** 10.1016/j.idcr.2021.e01135

**Published:** 2021-04-26

**Authors:** Aase Berg, Heidi Syre, Christine H. Ophaug, Jannicke H. Møller, Gry I.N. Behzadi

**Affiliations:** a Stavanger University Hospital, Department of Infectious Diseases, PO Box 8100, 4068, Stavanger, Norway; b Stavanger University Hospital, Department of Microbiology, PO Box 8100, 4068, Stavanger, Norway; c Stavanger University Hospital, Department of Intensive Care, PO Box 8100, 4068, Stavanger, Norway; d Stavanger University Hospital, Department of Radiology, PO Box 8100, 4068, Stavanger, Norway

A previous healthy 36-year-old woman presented to the emergency department of Stavanger University Hospital in August 2020 after five days with increasing neck pain. On admission, she had vomiting and severe mental confusion. She described acute impaired bilateral vision and right arm numbness, but general physical and neurological examination was remarkable normal. She had pulse 111 beats/minute, but otherwise normal vital signs, including no fever. Laboratory values showed leukocytosis with white blood cells 13,500/μL (normal range 3.900–10.400) and se-glucose 115 mg/dL (82–140). Other laboratory exams were within normal range.

The acute history with mental confusion, loss of vision and arm numbness without fever, raised suspicion of a cerebrovascular accident. Cranial CT and cerebral angiography were normal. Electroencephalogram showed moderate nonspecific, diffuse abnormities mostly bilateral fronto-temporally. Lumbar puncture done under general anesthesia, because of severe confusion and lack of cooperation, showed clear and normotensive cerebrospinal fluid (CSF). White blood cells were 634 cells/μL (<5), whereof 99 % monomorph-nuclear cells, protein 303 mg/dL (23–38) and glucose 39.6 mg/dL(<0.4 of se-glucose). Due to short history duration and low CSF glucose, early bacterial infection could not be excluded, even if a viral etiology was more probable. She was observed in droplet precaution isolation, and given ceftriaxone 4 g daily and acyclovir 750 mg three times daily, both intravenously.

MRI showed pathological contrast enhancement along several of the cranial nerves (Fig. 1, Fig. 2, Fig. 3, Fig. 4), even if a bit blurred due to confuse patient. Blood and CSF cultures were negative. FilmArray meningitis/encephalitis panel (BioFire Diagnostics) detecting the fourteen most common microbes associated with CNS infections were negative. The same applied to inhouse RT-PCR for Herpesvirus 1, Herpesvirus 2, Varicella zoster and Enterovirus. There were high levels of *Borrelia burgdorferi* IgM and IgG (175.9 and 106.7 AU/mL, respectively; Liaison, Diasorin) in serum, and even higher in CSF, with IgG index CSF/ serum (IDEIA, Oxoid) 19.5 and IgM index CSF/ serum 20.0 (normal ranges < 0.3).

**Fig. 1:**
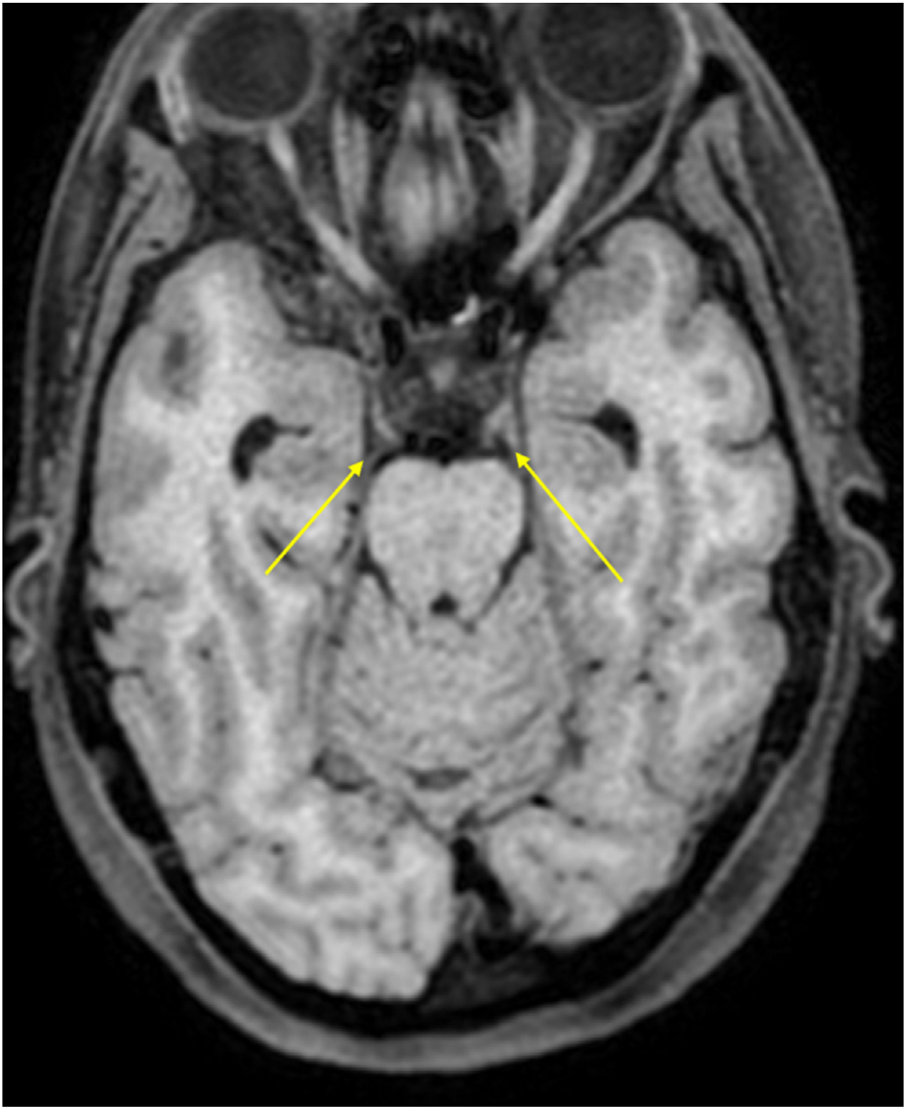
Oculomotor nerves (yellow arrows). Axial non-enhanced T1 weighted image.

**Fig. 2:**
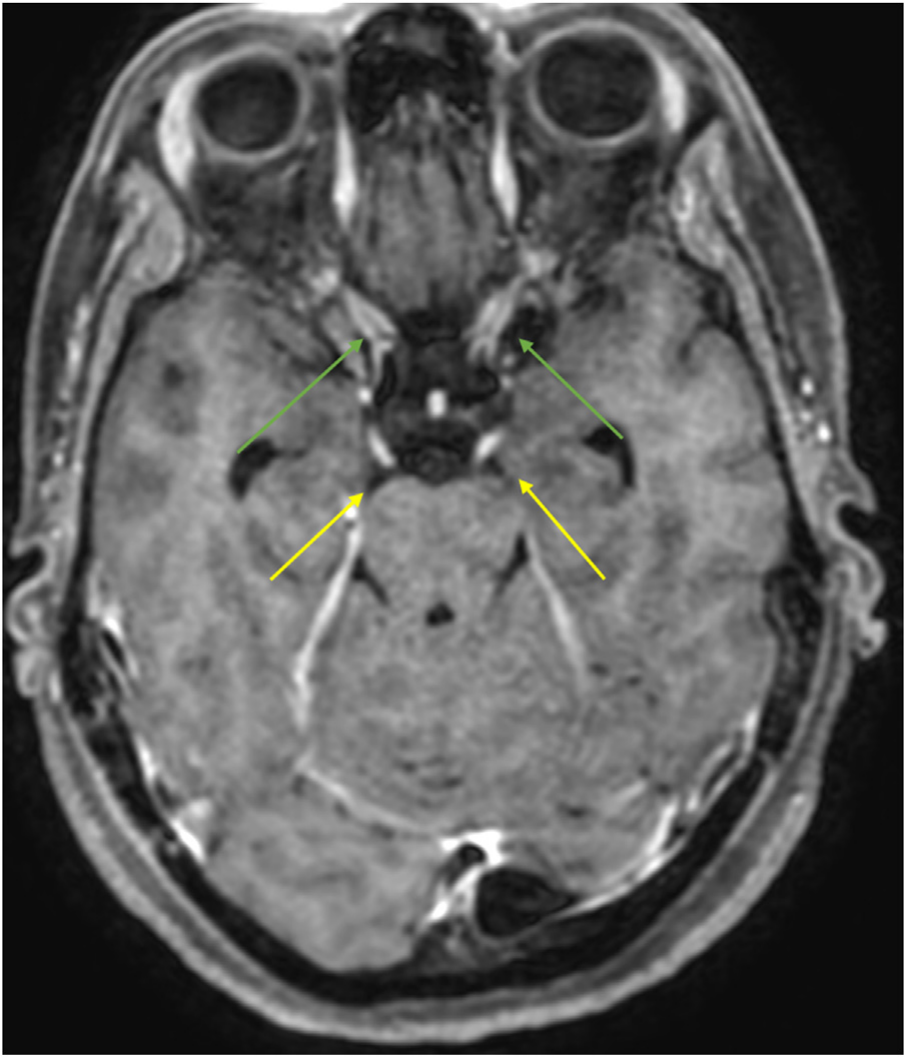
Enhancing oculomotor nerves (yellow arrows). Note that the optic nerves also enhance (green arrows). Axial Gd-enhanced T1 weighted image.

**Fig. 3:**
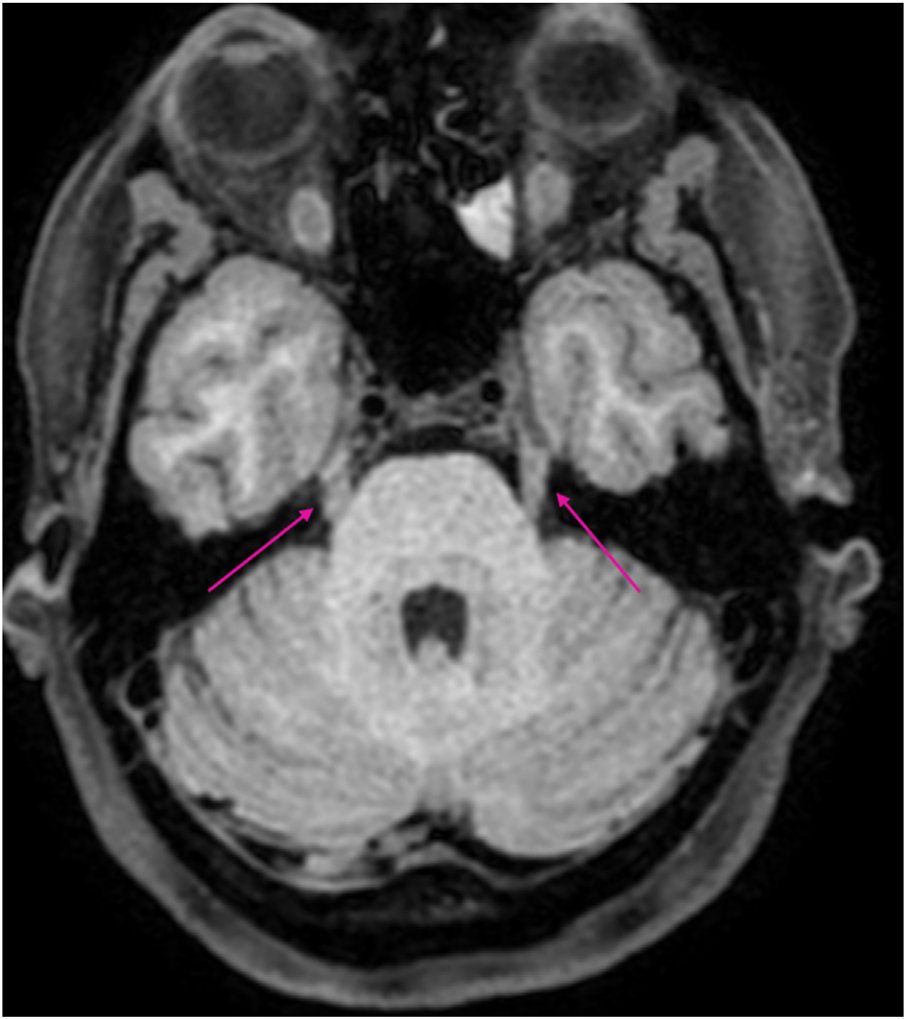
Trigeminal nerves (pink arrows). Axial non-enhanced T1 weighted image.

**Fig. 4:**
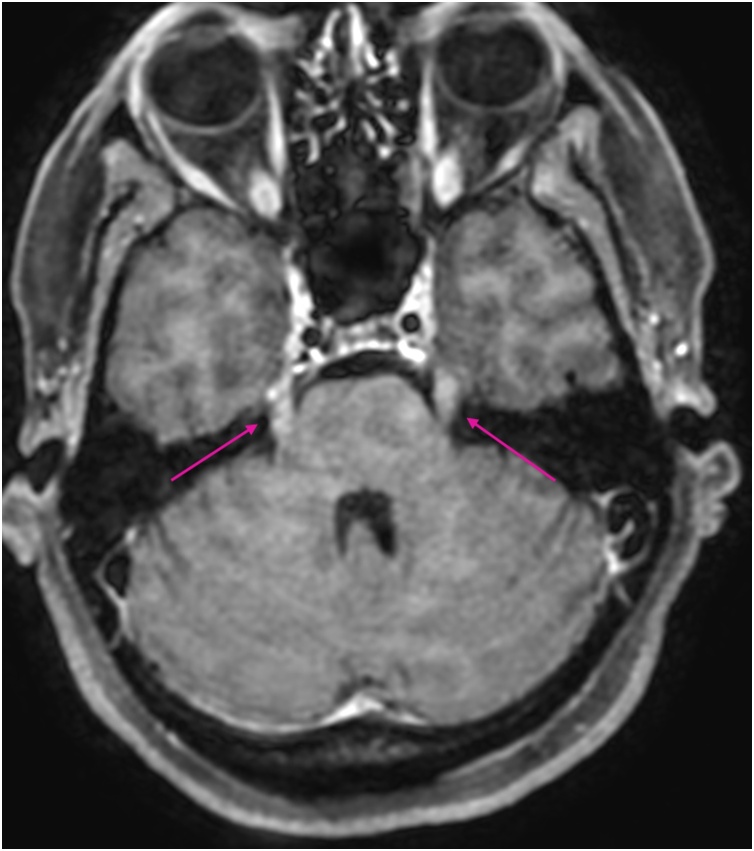
Enhancing trigeminal nerves (pink arrows) Axial Gd-enhanced T1 weighted image.

She was diagnosed acute nevroborreliosis and received ceftriaxone 2 g twice daily for three weeks. On follow-up four more weeks later, she had recovered well without any sequelae.

Enhancement of multiple cranial nerve in a T1-weightened MRI-scan, indicate cranial neuritis due to Lyme disease and is otherwise unusual [1,2]. It may be particularly useful when investigating a diffuse clinical presentation for differential diagnoses, as a supplement to lumbar puncture [3].

## Author contribution

Treatment and investigation of the patient, writing of the manuscript: ÅB, CHO, JHM Microbiological testing and interpretation, writing of the manuscript: HS Clinical pictures and the interpretation, writing of the manuscript: GINB

## Funding

There have been no funding sources.

## Ethical approval

Not applicable.

## Consent

The patient have given consent for the publication both orally and in written.

## Declaration of Competing Interest

All authors declare that they have no conflicts of interest.
